# In-vitro diagnosis of single and poly microbial species targeted for diabetic foot infection using e-nose technology

**DOI:** 10.1186/s12859-015-0601-5

**Published:** 2015-05-14

**Authors:** Nurlisa Yusuf, Ammar Zakaria, Mohammad Iqbal Omar, Ali Yeon Md Shakaff, Maz Jamilah Masnan, Latifah Munirah Kamarudin, Norasmadi Abdul Rahim, Nur Zawatil Isqi Zakaria, Azian Azamimi Abdullah, Amizah Othman, Mohd Sadek Yasin

**Affiliations:** Centre of Excellence for Advanced Sensor Technology, Universiti Malaysia Perlis, Perlis, Malaysia; Institute for Engineering Mathematics, Universiti Malaysia Perlis, Perlis, Malaysia; Nara Institute of Science and Technology, Takayama-cho, Ikoma-shi, Nara Japan; Department of Microbiology|, Hospital Tuanku Fauziah, Jalan Kolam, Kangar, Perlis Malaysia

## Abstract

**Background:**

Effective management of patients with diabetic foot infection is a crucial concern. A delay in prescribing appropriate antimicrobial agent can lead to amputation or life threatening complications. Thus, this electronic nose (e-nose) technique will provide a diagnostic tool that will allow for rapid and accurate identification of a pathogen.

**Results:**

This study investigates the performance of e-nose technique performing direct measurement of static headspace with algorithm and data interpretations which was validated by Headspace SPME-GC-MS, to determine the causative bacteria responsible for diabetic foot infection. The study was proposed to complement the wound swabbing method for bacterial culture and to serve as a rapid screening tool for bacteria species identification. The investigation focused on both single and poly microbial subjected to different agar media cultures. A multi-class technique was applied including statistical approaches such as Support Vector Machine (SVM), K Nearest Neighbor (KNN), Linear Discriminant Analysis (LDA) as well as neural networks called Probability Neural Network (PNN). Most of classifiers successfully identified poly and single microbial species with up to 90% accuracy.

**Conclusions:**

The results obtained from this study showed that the e-nose was able to identify and differentiate between poly and single microbial species comparable to the conventional clinical technique. It also indicates that even though poly and single bacterial species in different agar solution emit different headspace volatiles, they can still be discriminated and identified using multivariate techniques.

## Background

Clinically, the microbiology of diabetic foot infection is very unique because it involves either Gram-positive, Gram-negative aerobic, or anaerobic bacteria [1]; whether caused by single or combination of bacteria (poly microbial) infection [2-4]. Single bacteria are the only one bacterial species isolated from multiple bacterial species on debridement of wound. Poly microbial species is the mixing of bacteria species that exist on wound infection. Usually, moderate to severe soft tissue diabetic foot infections are poly microbial containing species such as *P. aeruginosa*, *S. aureus* and group of Enterococci. It usually occurs when the patients received empiric antibiotic therapy [5]. Therefore, selecting appropriate antibiotics for the treatment of diabetic foot infection is crucial. It requires careful consideration in terms of severity of infection, duration of wounds and previous antibiotic exposure [5-7]. Although, there is no data to suggest that speeding the diagnosis of diabetic foot infections by 2 to 3 days will improve patient outcomes, nevertheless, the proposed e-nose technology can also improve patient care by improving or reducing drug resistance to infection and more economical since it allows the use of narrow spectrum antibiotics [5,8,9].

In today’s clinical practice, diabetic foot infection is diagnosed and monitored through many techniques such as ulcer swabs, curettage of the ulcer base, and needle aspiration after normal saline injection [10-12] to determine the appropriate antibiotics treatment. Other techniques, including tissue biopsy obtained at the bedside or by resection at the time of surgery [11,13], may pose a risk to the patients since they involve surgical procedures and need appropriate care. Although these tests have been internationally standardized and are generally considered to be reliable, results still take 2 or 3 days. This constraint is due to the need to grow samples in media culture for at least 48 to 72 hours in order to identify bacteria species.

The use of an e-nose, such as the Cyranose320 to identify bacteria species may outperform conventional methods and address current limitations by providing a faster diagnosis. As a comparison, a chromatography technique that relies on a total ion chromatogram (TIC) may not be sufficient as volatile peaks can overlap [14-16]. However, GC-MS commonly applied for similar methods and mass separation can resolve peaks that are not separated by chromatography alone.

Alternatively, E-nose configured on an array of 32 different mixtures with conducting carbon black polymer sensors on a silicon substrate, was believed can be used to detect even the slightest difference in headspace or complex volatiles organic compounds (VOCs) emitted by the different pathogenic microorganisms [17-19]. As such, this non-invasive method may be promising for rapid and accurate detection. It can also prevent complications from a procedure, such as infection and contamination.

Several articles have reviewed clinical applications whereby e-nose technology was applied to non-invasive monitoring of patients [20] in various applications such as in clinical microbiology and for rapid diagnosis of infection from biological samples [18]. Moreover, there were at least 12 reported findings on the ability of an e-nose to identify and discriminate single bacterial species in a closed-loop system [19,21-31]. Previous studies have shown the ability and the robustness of an e-nose to detect the single strain of bacteria on blood culture medium [32,33], and hence opens the way toward making the e-nose applicable in further investigations by direct sniff to the samples [34-39]. Single-strain bacteria are accompanied by or produce characteristic odours often known as a surrogate parameter, and recognition of these odours can provide diagnostic clues, which in turn may aid in planning for early appropriate treatment.

There were many other analytical techniques that have been used for identification of VOCs emitted from bacterial such as solid phase micro extraction-MS (SPME-GC-MS) [40,41], proton transfer reaction-MS (PTR-MS) [42], GC with a flame-ionization detector (GC-FID) [13], selected ion flow tube-MS (SIFT-MS) [43], secondary electrospray ionization mass spectrometry (SESI-MS) [44], matrix-assisted laser desorption/ionization time-of-flight mass spectrometry (MALDI-TOF-MS) [45]. Novel basic odour detection was developed and targeted for poly and single microbial bacteria using direct injection of a static head space and in combination with multi-class odour recognition for robust detection of bacterial species on different agar mediums.

In this study, the SPME-GC-MS was chosen to determine the VOCs result since combination of both SPME and GCMS is very popular. SPME uses a polymer-coated fiber to concentrate volatile and semi-volatile organics in one extraction step [46,47]. Gas chromatography- mass spectrometry (GC-MS) is a method that combines the features of gas–liquid chromatography (GC) and mass spectrometry (MS) to identify different kind of substances within a test sample [48]. The key marker volatiles were detected and the volatile compounds emitted from wounds, where bacteria can often be found, verified the possibility of early recognition involved in infection using the SPME technique combined with gas chromatography–mass spectrometry (GC-MS).

To the best of the author’s knowledge, this research paper presents a novel work on the identification of both poly and single bacterial subjected to different agar medium using e-nose. Poly microbial infections of diabetic foot patients have not been well reported and documented as the research in this area is quite challenging. Thus, the significance of this experiment and the potential use of this application in a clinical setting are discussed.

## Methods

### Media culture preparation

Blood agar medium was prepared using a 20 g Tryptic Soy Blood Agar Base (TSBA) in 500 mL distilled water. The medium was then sterilized at 121°C and 225 kPa for 15 minutes and cooled to room temperature. After that, 25 mL of sterile defibrinated blood was placed in the medium and stored at 4°C.

The same procedure was also applied to the MacConkey agar using 25 g powdered MacConkey in 500 mL of distilled water. The medium was then heated with frequent agitation and boiled for one minute to completely dissolve. After the medium was autoclaved at 121°C for 15 minutes, the prepared media may turn to dark pink and trace to slightly hazy.

The Mueller Hinton agar was prepared by suspending 19 g of the medium in 500 mL of purified water. The medium was heated with frequent agitation and left to boil for one minute to completely dissolve. After the medium was autoclaved at 121°C for 15 minutes, it appears as hazy and light to medium yellow when cooled to room temperature.

### Bacteria isolation

Bacterial isolates obtained from the samples debridement of diabetic foot wound (wild-type bacteria) and American Type Culture Collection (ATCC) standard bacterial was prepared as shown in Table 1. Informed consent was obtained from all patients. This method has been granted approval by the *University Research Ethics Committee*, Universiti Malaysia Perlis to diagnose of single and poly microbial species targeted for diabetic foot infection using e-nose technology. ATCC bacteria are a commercially available bacterium that is used as a standard reference in research. The isolation of bacteria was divided based on gram positive and negative of bacteria strains. Each of the samples was prepared in five plate culture with three different mediums. The total number of data analysis represents the five times repeated measurements of data collected by using the e-nose. The bacterial suspension solution was adjusted to a turbidity of a standard Mac Farland 1.0 (3 × 10^8^ cfu/mL) in a NaCl solution and then subculture on three different media based on the gram strain of the bacteria. The bacteria were then incubated at 37°C for 24 hours before analysis.

**Table 1:** Single and poly microbial species culture on different medium

	**Total no. of data analysis collected by e-nose**		
**Organism**	**Blood agar**	**Mueller Hinton**	**MacConkey**
Gram positive aerobes			
* S. aureus ATCC 29213*	750	750	-
S. aureus*	500	500	-
Gram negative aerobes			
* E. coli ATCC 35218*	750	750	750
E. coli*	500	500	500
* P. aeruginosa ATCC 27853*	750	750	750
P. aeruginosa*	500	500	500
Mix gram negative aerobes			
E. coli* + P. aeruginosa*	350	350	350
*Mix gram positive and negative aerobes*			
E. coli* + S. aureus*	350	350	-
S. aureus* + P. aeruginosa*	350	350	-

*Isolated from debridement of diabetic foot wound (wild-type bacteria).

### E-nose and odour sniffing setup

The human olfactory system (sense of smell) is a very complex process and responds to certain volatile chemicals that are thought to be important in the detection of irritants and chemically reactive species [17]. Cyranose320 was selected as an effective instrument that “electronically” mimics the human olfaction system. It was developed as a simplified model of the human olfactory system and was designed to detect and discriminate different complex odours based on a sensor-array concept.

**Figure 1:**
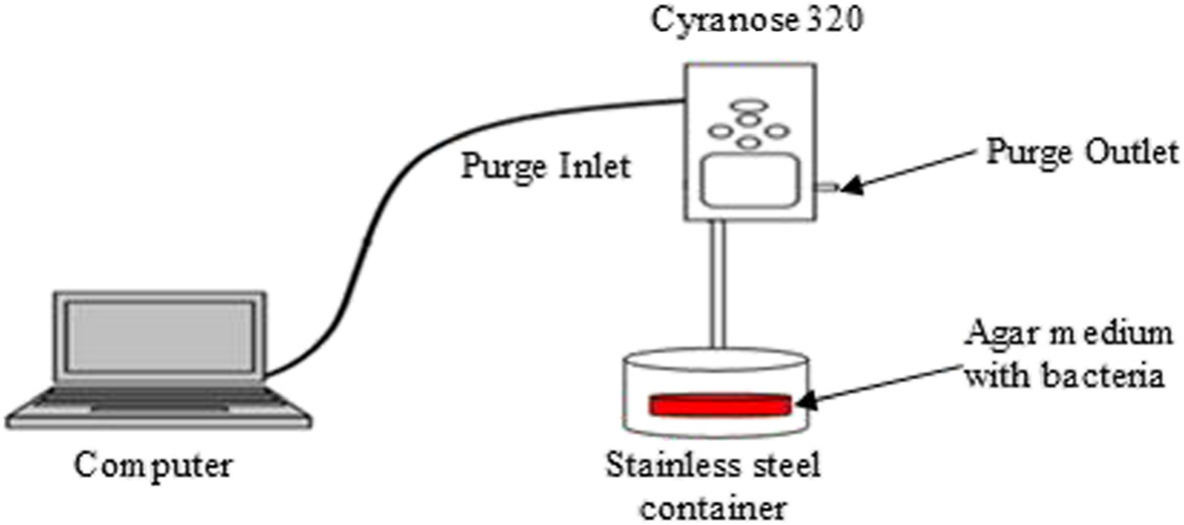
Odour sniffing setup. Cyranose320 (e-nose) was setup for headspace evaluation of poly and single bacteria species infusions.

This sensor array consists of broadly tuned (non-specific) and highly selective sensors that are coated with a variety of odour-sensitive absorbent materials. The array combinations together with the odour sniffing setup allow the e-nose to be tuned for a specific application, for example to detect slight changes in an odour profile. The e-nose setup is shown in Figure 1.

### Sampling procedure and detection system

The sampling procedure is started after bacteria were incubated for 24 hours. The investigation was performed on both bacteria (wild-type and ATCC standard) in different nutrient medium, incubated for at least 24 hours to project headspace variability, which may be emitted by the bacterial infection of the patients. A Cyranose320 e-nose system was used to sniff all the samples by transferring the headspace of target samples to the sensors without altering its composition and properties. The target medium agar was inserted in a closed container and the odour sniffing started immediately. This procedure was implemented in such a way as to ensure consistency in e-nose performance on immediate sniffing (in-vivo) of the patient’s wound at the clinical stage later. Table 2 shows the measurement parameters for the Cyranose320.

**Table 2:** The parameter setting for poly and single bacteria assessment

**Cycle**	**Time (s)**	**Pump speed**
Baseline purge	10	120 mL/min
Sample draw	60	120 mL/min
Idle time	3	-
Air intake purges	40	160 mL/min

The analyses result from the use of the e-nose was compared to the results with SPME-Gas Chromatography Mass Spectrometry (SPME-GCMS) to validate the obtained data. Conventional multivariate analysis such as LDA is shown to work effectively with a lower number in class variability. In order to ensure robustness, e-nose data which comprises of high number of class variability was then subjected to different classifiers such as KNN, SVM, and PNN. A hybrid LDA-Classifier was introduced to enhance prediction capability and the performance was compared with classical neural network and statistical methods. These multi-class classification techniques applied in this study was aimed to determine the best classifier to achieve the research objectives.

### SPME and GC-MS setting

A method based on the use of headspace, solid phase micro extraction (SPME), and GCMS was developed to determine the VOCs produced by a single or poly microbial infection extract from patients. The SPME technique was applied using a fused silica-fiber coated on the surface with a film of an immobilized stationery phase that is connected to the plunger of a modified GC syringe and moves inside the needle. The needle made by CAR/PDMS (Supelco-57320-U, Bellefonte, PA, USA) was used in this study to extract the volatile emitted from the bacteria. Then, the needle which has been exposed to the sample headspace for 10 minutes is injected into the manual injector port of a gas chromatograph system.

### SPME-GC setting

An SPME fiber (75 lm Carboxen-PDMS; Supelco, Inc., Bellefonte, PA, USA) was exposed to the sample headspace for 10 minute. The Volatile Organic Compound (VOC) was desorbed by inserting the SPME fiber into a GC injector (injector temperature 230°C) in split less mode connected with a fused-silica GC column (Elite 5MS, 30 m, 0.25 mm ID, 0.25 μm film thickness) (Perkin Elmer, Shelton, USA) for 10 minute. The initial temperature of the GC was set at 70°C for 0.5 min, and then the oven temperature was increased at a rate of 20°C/min until it reaches 250°C which remained for another 1 min. The detector temperature was set at 250°C.

### GC-MS setting

For GC-MS analysis, a GC (Clarus680) coupled with a mass spectrometry (Clarus600T, Perkin Elmer, Shelton, USA) was used. The GC operating conditions (temperature and time) were the same as described above. The mass spectrometer was operated in the electron-ionization (EI) mode at an ionization voltage of 70 eV.

In order to support the findings of this research, output from GC-MS were also analysed using LDA, KNN, SVM and PNN. Usually GC-MS provides information on specific analytes of interest (selective ion monitoring (SIM) and mass spectra data (SIM and total ion current (TIC). In this research, TIC data was used as input for the classifier. TIC is merely the sum at each time point of every mass-to-charge ratio (m/z) value across a mass spectrum [14].

### Data analysis and odour recognition

**Figure 2:**
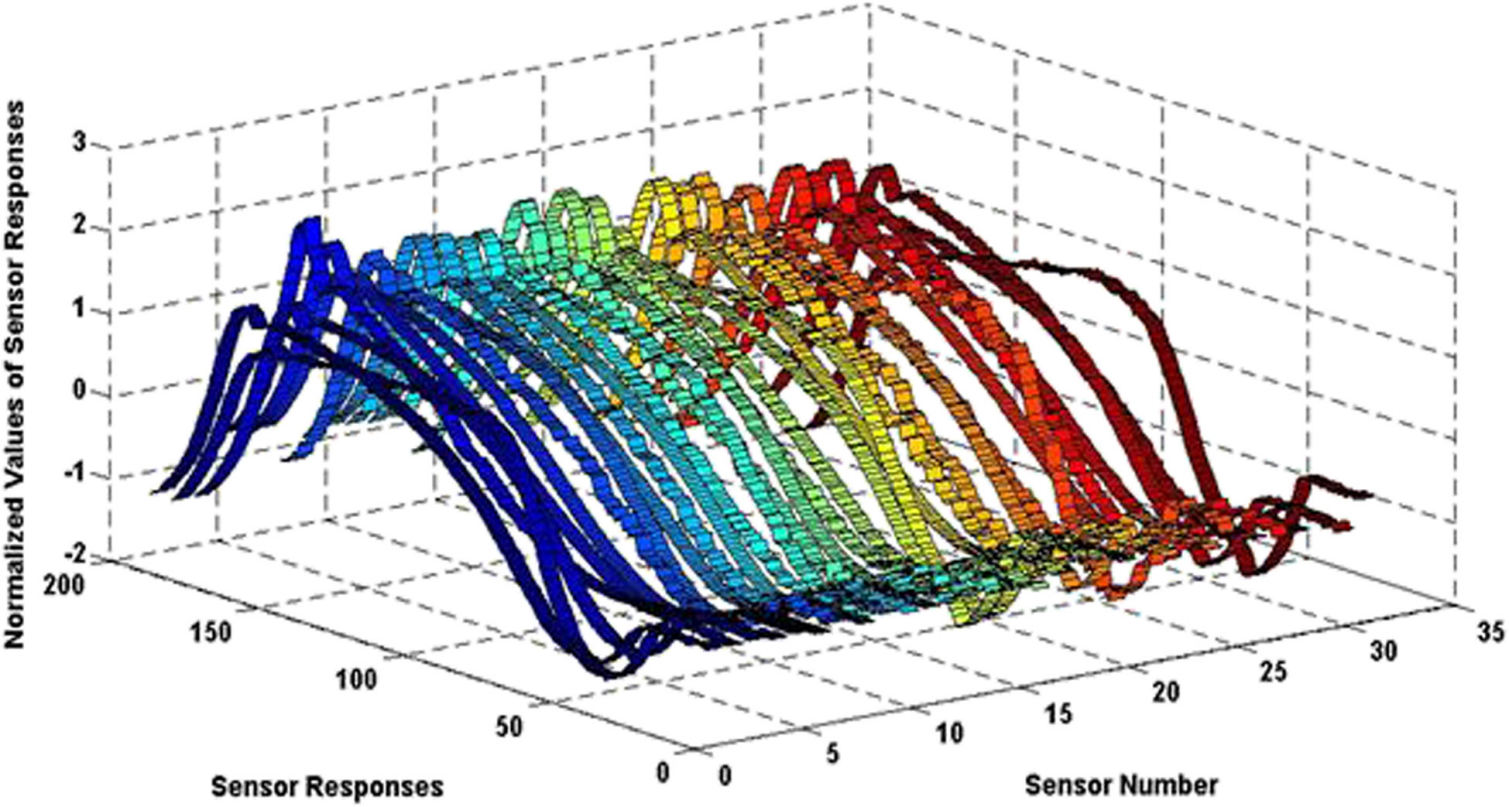
Signal response for odour recognition. A surface plot shows the visualized data for single bacteria samples gathered using a Cyranose320 e-nose system.

A surface plot was used to visualize the signal response from the e-nose in a sensor array configuration using the standard toolbox “surfc” functions of MATLAB R2013a to generate graphical plots of all 32 sensors during sniffing. This scientific software is normally used for signal processing [49]. The surfc () function was used to view mathematical functions over a rectangular region and to create colored parametric surfaces specified by normalized values of the sensor responses, sensor number, and the number of gathered data points as shown in Figure 2.

The response pattern of the sensor array is known as a ‘smell print’ and different bacteria may exhibit different smell print patterns as shown in Figure 2. For instance, sensor 5 is the most sensitive (emitting the highest response) compared to other sensors towards the volatile odour produced by *E. coli* bacteria. Both sensor 2 and sensor 32 showed the lowest response and may be less sensitive to volatile compounds produced by bacteria.

To ensure the consistency and the robustness of the e-nose, the dataset was also subjected to different classifiers such as KNN, SVM, and PNN. The performance of each classifier is discussed and compared. The Wilks’ Lambda statistical test was carried out to evaluate the statistical significant of all 32 independent conducting polymer sensors by comparing means score of 7 different groups samples, simultaneously. A multi-class odour classification model was later proposed to evaluate the robustness of an e-nose system in classifying unknown single and poly microbial samples.

### Feature extraction and dimension reduction

In this experiment, the steady-state response of each sensor during the sniffing phase was used as a feature extraction. The sensor response was determined by obtaining the difference in values between the ends of the sampling values with the baseline values, which can be denoted as feature extraction. This baseline correction is required to remove a ‘drifting effect’.

Principal Component Analysis which is an exploratory data analysis was used to extract the most influential sensors based on the highest principal component. Later, the dataset is fed through a linear classifier known as linear discrimination analysis (LDA) to quantify the discrimination performance of the e-nose. LDA was selected to reduce high-dimensionality data into a much lower dimension and to maximize class separability for bacterial identification [50].

## Results

### E-nose results

**Figure 3:**
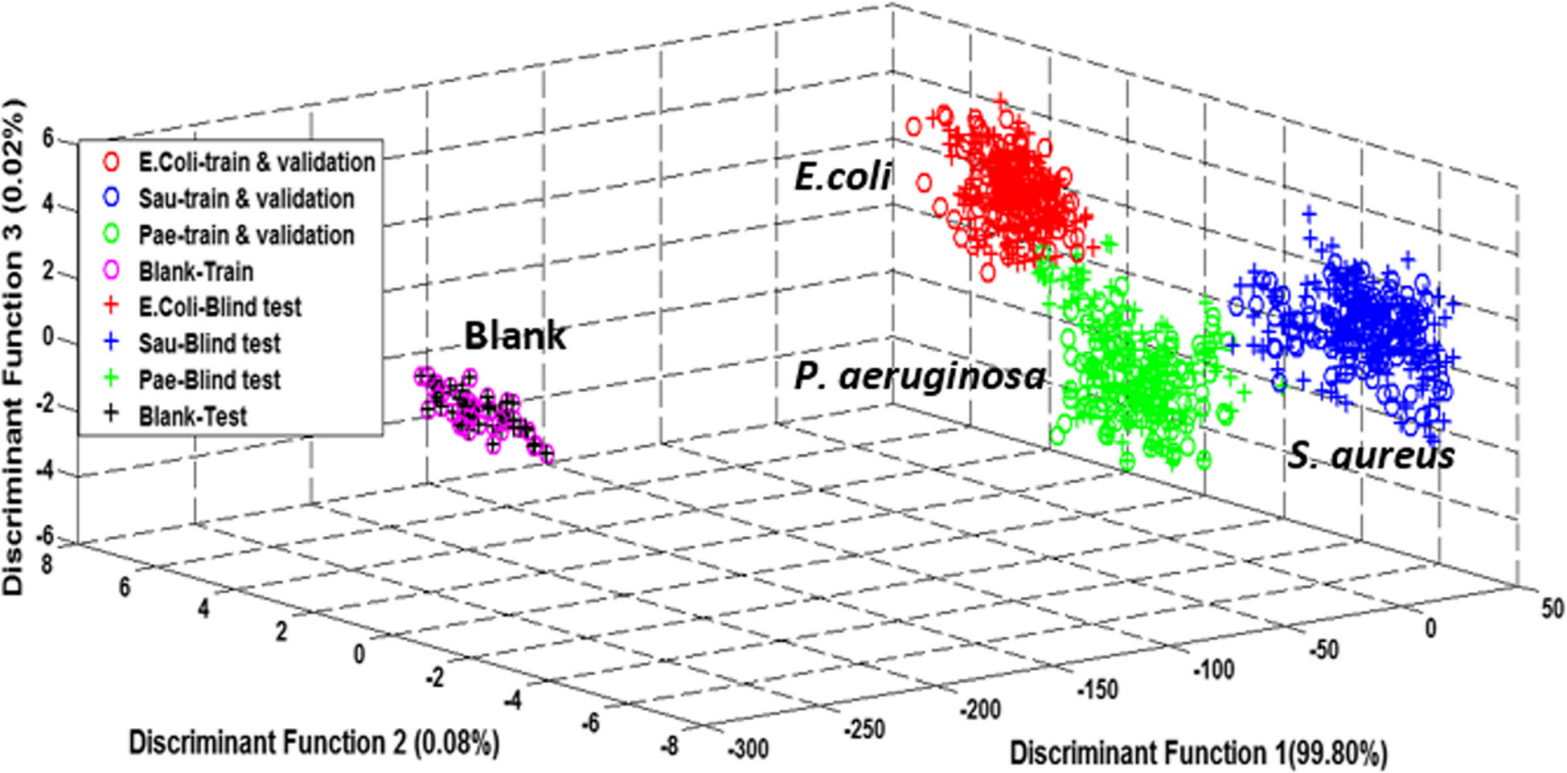
Supervised LDA plot for ATCC standard and wild-type bacteria in blood agar. The wild-type bacteria obtained from the samples debridement of diabetic foot wound were compared with the ATCC standard bacteria. The sign ‘+’ and ‘O’ in the plot are used to highlight the training and testing phase (blind test).

The preliminary work was focused on the investigation of volatiles released by a single bacterial species. This also included one-to-one comparison between a wild-type strain and standard ATCC bacteria combined in a blood agar medium. Three different species of bacteria namely, *E. coli, S. aureus*, and *P. aeruginosa* obtained from ATCC standard were cultured and the volatiles released were measured after an incubation process of 24 hours at 37°C. Figure 3 shows the results obtained by supervised LDA. The most striking finding was a clear separation between all three different bacteria when LDA was applied.

The result shows that each bacteria species does emit a distinct smell including the blind test that includes wild-type bacteria. Moreover, the blind test samples matched closely with the distribution of the different groups of bacteria in the training data. Both the wild-type strain and ATCC standard bacteria of the same species are clustered in the same group, showing that they emit the same volatiles. Distinct separations between group samples were observed even when the groupings of different bacteria were close to each other. This proves that the e-nose was able to discriminate different volatiles emitted from ATCC and wild-type bacteria.

**Figure 4:**
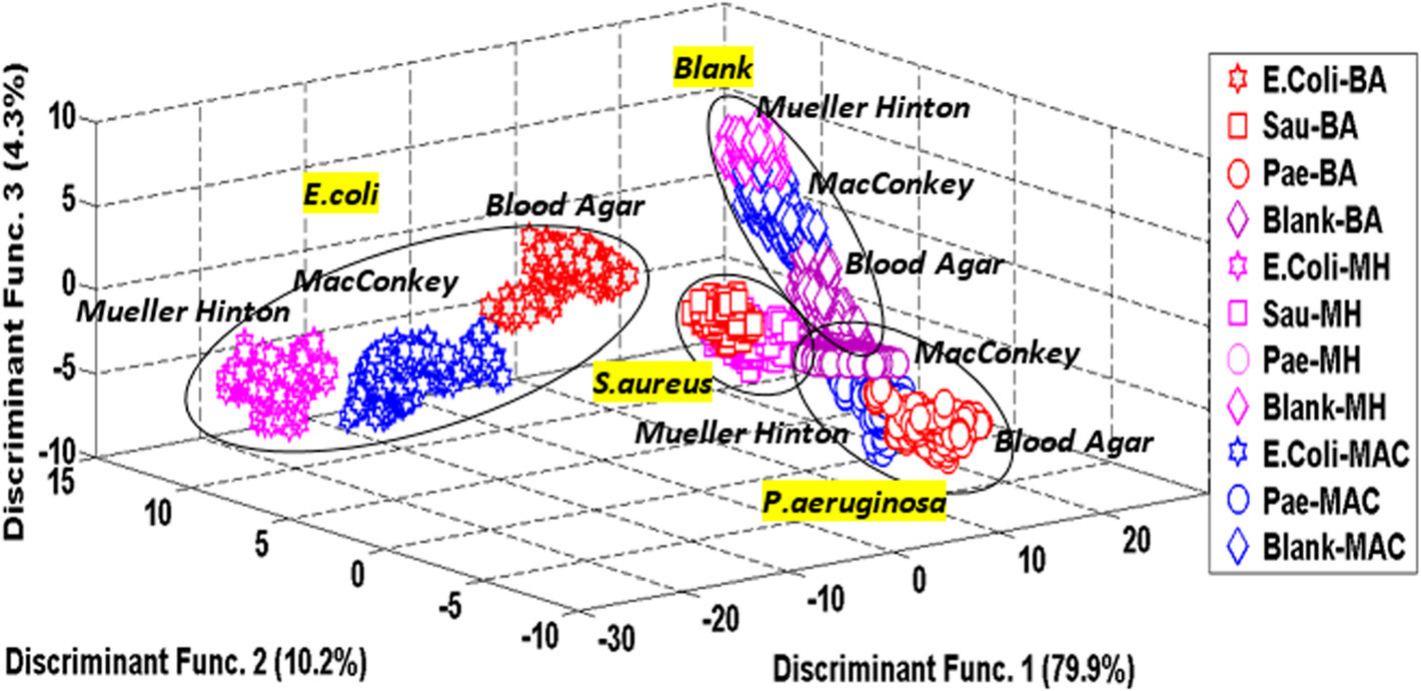
LDA plot of single bacteria species in three different mediums. This diagram is to show the clear vision of one group of single bacteria in three different mediums (blood agar, Mueller Hinton & MacConkey).

Further studies was conducted to extend the capability of e-nose to identify more groups of different bacteria categorize under single and poly microbial species within different mediums. Single and poly microbial species as described in the background are investigated and classified using LDA, PNN, KNN and SVM. Due to the limited ratio of the classification diagram and unclear classification plot, the analysis was carried out for both groups of bacteria. However, to clearly show the classification result, one of the diagrams is illustrated in Figure 4.

**Figure 5:**
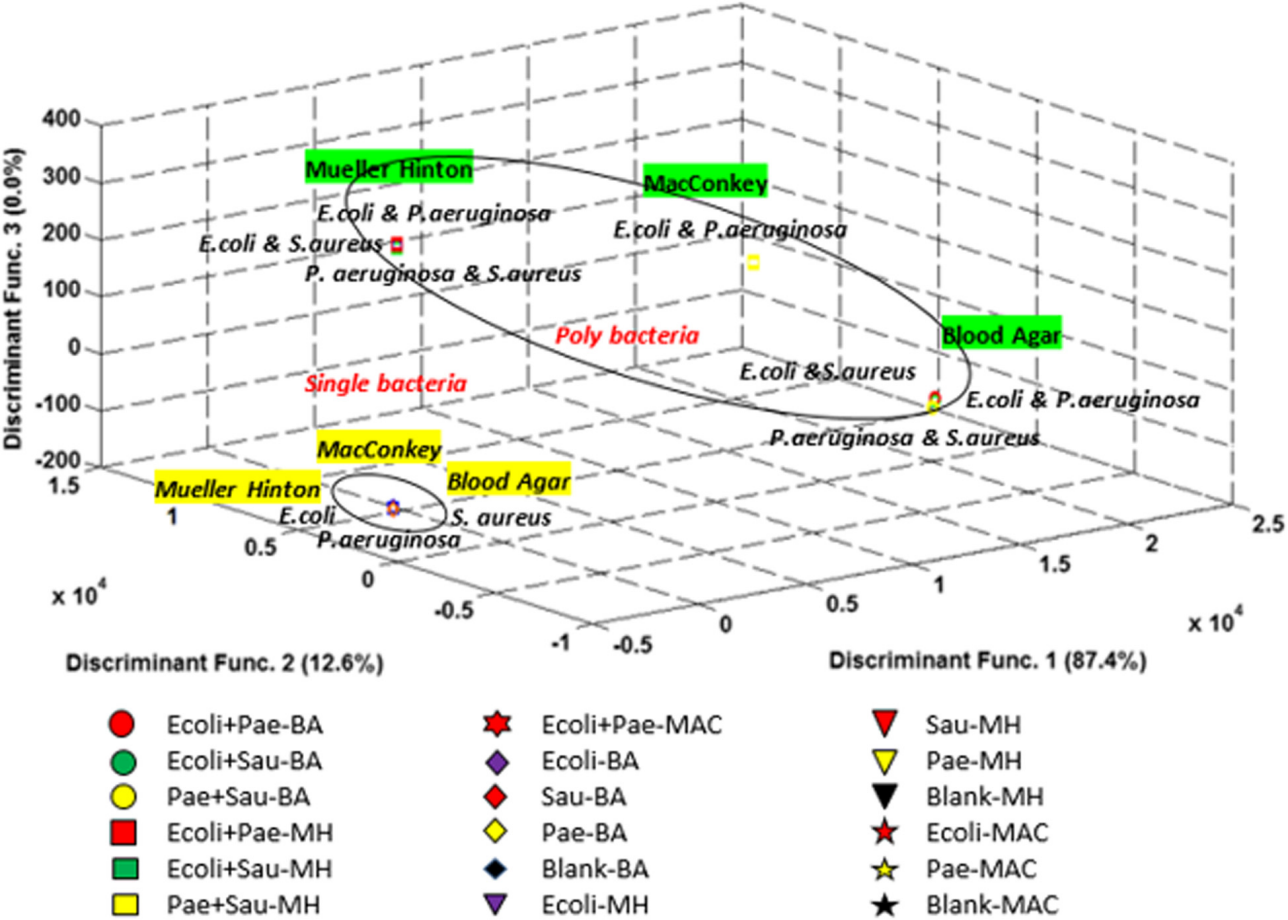
LDA plot of single and poly microbial species in three different media. This combination of single and poly microbial in different media is to study the effectiveness of e-nose to classify the bacteria in a different group region.

Figure 4 shows the classification of single bacteria that has been separated into their groups. While Figure 5 shows the overall classification result of both groups of bacteria.

Figure 5 shows the two groups of single and poly microbial species that were successfully classified. For different types of single and poly microbial species dataset, eighteen groups (G = 18) were involved with 32 features (*p* = 32) of the e-nose. The number of useful discriminant functions that can separate the bacteria species by different mediums is the minimum of (G-1) or *p*, and in this case it is the minimum of 17 and 32, that is 17. In this case, a maximum of 17 useful discriminant functions (DF) can be applied to separate the bacteria species. However, for display purposes only the highest discriminant functions are considered to show the actual classification among 18 different groups of bacteria. Among the highest discriminant function accounted for display are 87.4% for DF1 and 12.6% for DF2.

On the other hand, the Wilks’ lambda test shows that, there is a statistically significant relationship between the independent variables and the group samples whereby the *p-value* of Discriminant Function 1 through 5 is less than 0.001 as shown in Table 3. The Discriminant Function 6 was statistically less significant as both p < 0.003 and Wilks’ Lambda value were high. Only the first 3 Discriminant Function was used in the latter analysis.

**Table 3:** The statistical significance classifiers using Wilks’ Lambda

**Test of function(s)**	**Wilks’ Lambda**	**Chi-square**	**df**	**Sig.**
1 through 6	0.000	19972.418	54	0.000
2 through 6	0.025	6706.844	40	0.000
3 through 6	0.180	3115.957	28	0.000
4 through 6	0.444	1477.723	18	0.000
5 through 6	0.760	499.888	10	0.000
6	0.991	15.784	4	0.003

### Evaluation and classification performance

A hybrid LDA-Neural Network classifier was introduced to enhance prediction of unknown sample. Table 4 shows the multi-class classification accuracy of single and poly microbial species when different sets of features are subjected to different classifiers. In order to estimate the performance of the classifier, we adopted the common “leave-one-out” cross validation technique to train and test the remaining samples which were not used during the cross validation process.

**Table 4:** Classification accuracy of both single and poly microbial species in three different mediums using different classifier

	**Blood agar (1,750 data)**		**Mueller Hinton (1,750 data)**		**Mac Conkey (1,000 data)**		**Mixed media (4,500 data)	
**Classifiers**	**Train & validation (733 data)**	**Test (1017 data)**	**Train & validation (733 data)**	**Test (1017 data)**	**Train & validation (399 data)**	**Test (601 data)**	**Train & validation (1792 data)**	**Test (2708 data)**
LDA	100%	100%	99.7%	98.7%	100%	100%	95.9%	94.7%
PNN	100%	100%	100%	89.7%	98.7%	98.2%	65.2%	63.3%
KNN	100%	100%	97.0%	95.4%	100%	100%	99.9%	99.2%
SVM	100%	100%	100%	100%	100%	100%	67.3%	65%
*PNN	100%	100%	100%	96.3%	100%	100%	99.2%	96.8%
*KNN	100%	100%	100%	100%	100%	100%	99.9%	99.6%
*SVM	100%	100%	100%	100%	100%	100%	98.9%	98.7%

*Data was extracted from a discriminant function of LDA and subjected to classifier input.

**Combination of three media (blood agar, Mueller Hinton & MacConkey), p value < 0.001.

The results show that almost all classifiers performances in these three different media (blood agar, Mueller Hinton and MacConkey) were achieved up to 89% accuracy. The blood agar sample was achieved 100% accuracy, using feature extraction and without feature extraction of the LDA, which indicates an excellent result compared with Mueller Hinton and MacConkey medium. While for PNN and SVM classifiers, using raw data, was not able to achieve higher classification of mixed media data samples. However, using data extracted from a discriminant function of LDA, performance of 96% accuracy was achieved. The hybrid LDA-KNN achieves highest classification accuracy in mixed media culture. Despite of some inconsistency that were observed in the classification bacteria species of mixed media, the e-nose is still a promising tool for *in-situ* identification of bacterial infection. Since the highest accuracies obtained for information extracted using LDA, so those data were applied to show the sensitivity and specificity for each bacteria species in mixed media as illustrated in Table 5.

**Table 5:** **Sensitivity and specificity of both single and poly microbial species in mixed media using different classifier**

		**Classifiers**							
**Mixed media culture**	**Bacteria species**	**LDA %**		**PNN %**		**KNN %**		**SVM %**	
		**Sensitivity**	**Specificity**	**Sensitivity**	**Specificity**	**Sensitivity**	**Specificity**	**Sensitivity**	**Specificity**
BA	Ecoli	100	99.65	100	100	100	100	100	100
	Sau	83.11	98.44	76.19	98.01	74.84	98.16	72.44	100
	Pae	98.72	99.96	100	100	100	100	87.08	100
	Blank	96.48	99.30	100	100	100	100	70.14	100
	Ecoli + Pae	100	100	100	100	100	100	85.88	100
	Ecoli + Sau	100	100	100	100	100	100	100	99.07
	Pae + Sau	100	100	100	100	100	100	100	100
MH	Ecoli	100	100	100	100	100	100	92.77	100
	Sau	75.76	99.02	69.28	98.62	70.25	98.47	77.19	97.61
	Pae	100	100	100	100	100	100	100	98.99
	Blank	87.50	99.84	98.68	99.96	99.34	99.96	81.18	100
	Ecoli + Pae	95.68	99.49	99.32	100	99.32	100	67.20	100
	Ecoli + Sau	100	99.80	100	100	100	100	100	98.51
	Pae + Sau	90.78	99.96	100	99.96	100	99.96	100	98.81
MAC	Ecoli	94.23	100	100	100	100	100	100	99.53
	Pae	96.03	99.73	100	100	100	100	100	98.46
	Blank	88.89	99.18	99.36	99.92	99.36	99.96	100	96.81
	Ecoli + Pae	100	100	100	100	100	100	100	99.96

BA: Blood agar; MH: Mueller Hinton; MAC: MacConkey; Ecoli: *E. coli;* Sau: *S. aureus;* Pae: *P. aeruginosa.*

Table 5 represents the sensitivity and specificity for different classifier for each of the bacteria species in mixed media culture. From the perspective of specificity, generally it can be concluded that all the classifiers are good at specifying the different bacteria species in different media culture with their actual group. However, the classifiers’ sensitivity is inconsistent where some of the bacteria species using LDA, PNN, KNN and SVM were concerned, was unable to detect some of the bacteria species with particular mediums into the correct groups. Although the performance of KNN classifier achieved the highest accuracies compared to other classifier, refer Table 4, this classifier has low sensitivity to differentiate *S. aureus* bacteria in blood agar and Mueller Hinton media.

### Headspace SPME-GCMS results

**Figure 6:**
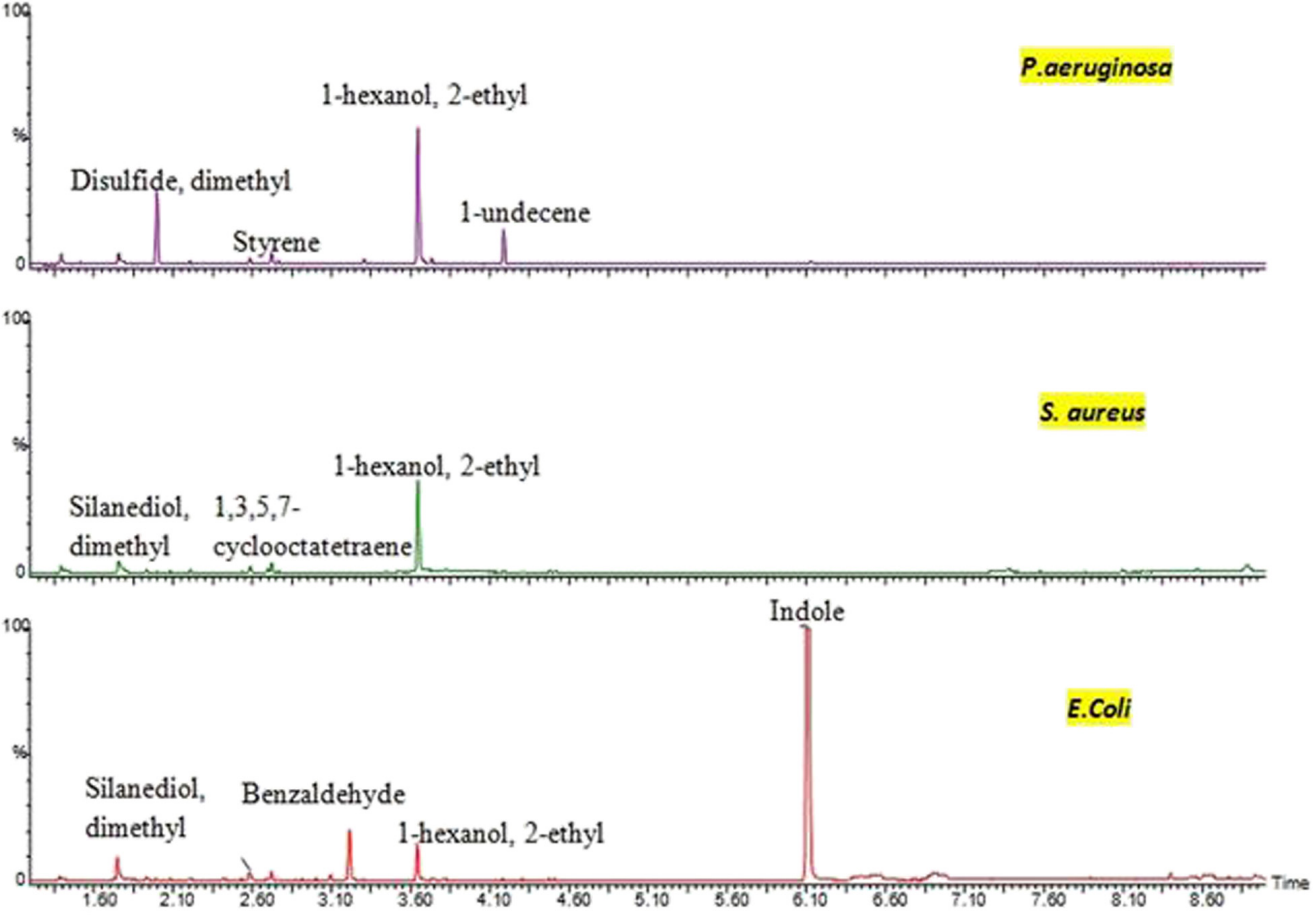
Chromatograms of single bacteria species using headspace SPME-GCMS. The GCMS result shows that the different bacteria species emitted different biomarkers which confirm the e-nose result.

**Figure 7:**
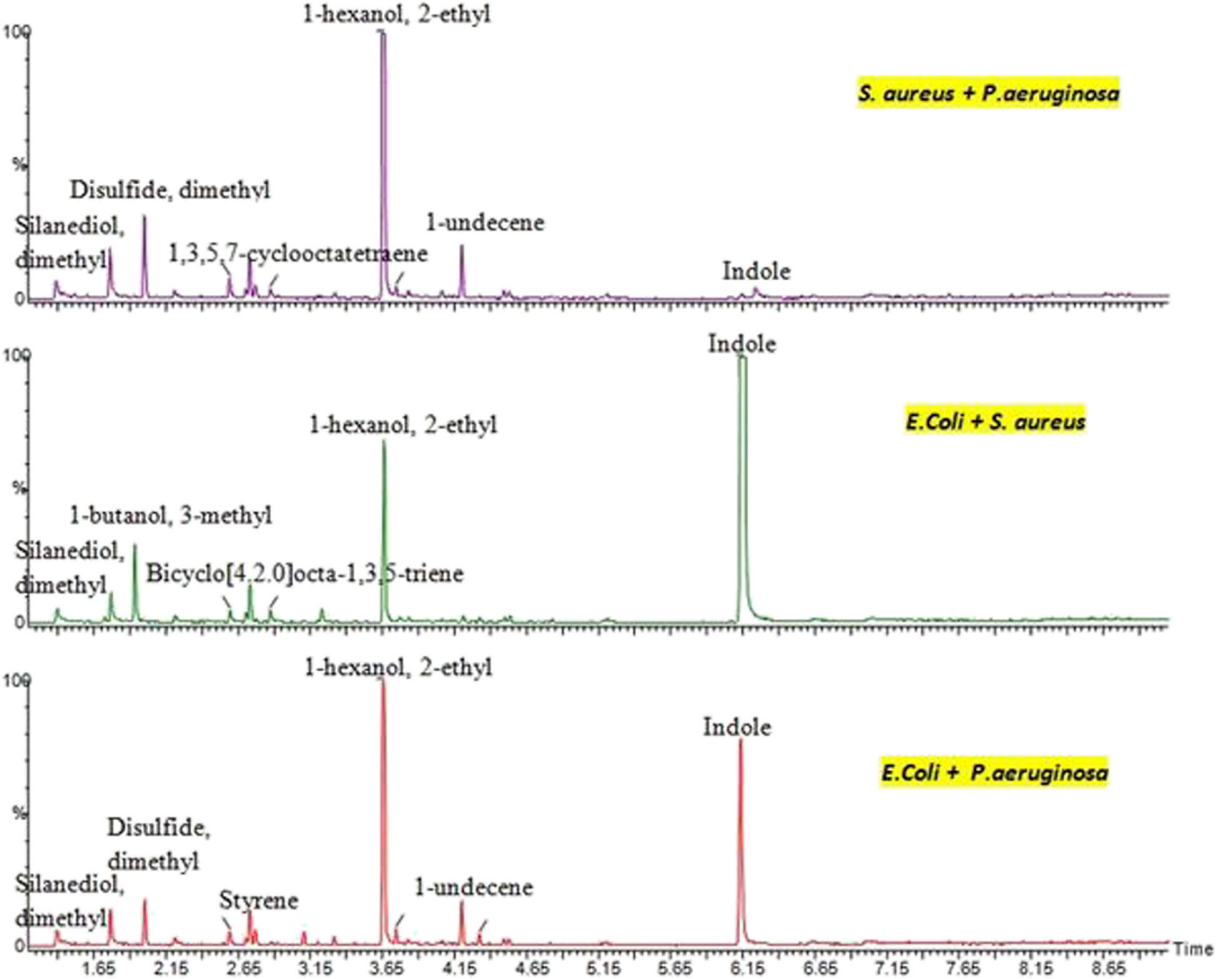
Chromatograms of poly microbial species using headspace SPME-GCMS. The GCMS result shows that the VOCs emitted when combine the two bacteria species in one media culture.

The total ion chromatogram (TIC) data of a single and poly microbial profile from headspace-GC-MS is illustrated as in Figures 6 and 7.

Every peak obtained was identified by matching sample mass spectrum with those of the National Institute of Standards and Technology (NIST) MS spectral library for peaks presented in the chromatograms. It has been observed that the biomarkers from ‘TIC peaks’ for single bacteria species can be extracted and used for classification. The TIC peaks clearly show the VOCs produced from that particular bacteria. Unfortunately for poly microbial species in Figure 7, the bio-marker peaks are overlapped. Intensive data collections on poly microbial samples are required and must be subjected to chemometric technique. This to ensure correct bio-markers of poly microbial profile can be extracted from the TIC spectrogram.

## Discussion

This paper proposed multi-class classification of single and poly microbial species *in vitro* to target diabetic foot infection. As mentioned previously in Figure 3, the wild-type bacteria obtained from debridement of diabetic foot wound produced almost similar VOCs with the standard ATCC bacteria. This proves that the wild bacteria species used in this experiment such as *E. coli*, *S. aureus* and *P. aeruginosa* have similar features with the standard ATCC bacteria as a reference. The e-nose was able to identify wild-type bacteria and match it with the standard bacteria samples.

Moreover, we preceded our investigations by analysing our data from a combination of the two groups of bacteria species in different media culture as shown in Figure 5. We found that the odour produced from single bacteria species was totally different with the odour produced by poly microbial species. This occurs because when two different gram-stain bacteria interact, they produce different volatile odours due to metabolic reactions to specific biochemical precursors and interactions among themselves while digesting nutrients [28].

The chromatograms from GC-MS allowed detailed analysis of potential biomarkers. Figure 6 shows an interesting result for three different bacteria species. *E. coli* emitted the highest peak of indole which showed similar results with other research studies [44,51]. Hence, the indole produced can be described as a common diagnostic biomarker for the identification of this bacterium.

It same goes with the TIC result for *P. aeruginosa* which consists of compounds undecene and methyl group (alkenes). Although, the highest peak was 1-hexanol-2-ethyl compound, 1-undecene and styrene can be concluded as a biomarker of *P. aeruginosa* since those compounds are only found in *P. aeruginosa* profile. This finding confirmed with studies of [51,52].

Furthermore, the results TIC for *S. aureus* also showed that it contains alcohol group (silanediol), disulphide dimethyl, 1, 3, 5, 7-cyclooctatetraene and the highest peak was 1-hexanol-2-ethyl. Based on the VOC produced, 1, 3, 5, 7-cyclooctatetraene was the biomarker for that bacteria. These biomarker can be confirmed with other research such as [44,52].

Unlike single bacteria groups, the combinations of mixed bacteria species as illustrated in Figure 7 create a unique smell print pattern for each bacterium. It consists of indole, methyl group and alcohol group such as hexanol and butanol. Those VOCs could be used as a volatile smell print of each bacterium. It has been observed that the biomarkers from the ‘TIC peaks’ for single bacteria species clearly showed the VOCs produced from that particular bacteria and can be extracted and used for classification. However, the GCMS result for poly microbial species are not conclusive since the bio-marker peaks are overlapped. Intensive data collections on poly microbial samples are required and later must be subjected to a chemometric technique which is not a straight forward analysis.

## Conclusions

In conclusion, the analysis on the e-nose system was brought one stage closer to medical application as our study used real patient samples, rather than pure laboratory cultures, but a culture stage was still involved. The sampling procedure was carefully formulated to serve as proof of concept that will enable further work on in-vivo (direct sampling) of bacterial infection on diabetic foot ulcers.
